# A scoping review of full-spectrum knowledge translation theories, models, and frameworks

**DOI:** 10.1186/s13012-020-0964-5

**Published:** 2020-02-14

**Authors:** Rosmin Esmail, Heather M Hanson, Jayna Holroyd-Leduc, Sage Brown, Lisa Strifler, Sharon E Straus, Daniel J. Niven, Fiona M. Clement

**Affiliations:** 1 Department of Community Health Sciences, Cumming School of Medicine, University of Calgary, 3D14A Teaching and Wellness Building, 3280 Hospital Drive NW, Calgary, Alberta T2N 4Z6 Canada; 2 Alberta Health Services, Calgary, Alberta Canada; 3 O’Brien Institute for Public Health, University of Calgary, Calgary, Alberta Canada; 4 Department of Medicine, Cumming School of Medicine, University of Calgary, Calgary, Canada; 5 Hotchkiss Brain Institute, University of Calgary, Calgary, Alberta Canada; 6 Health Technology Assessment Unit, University of Calgary, Calgary, Alberta Canada; 7 Li Ka Shing Knowledge Institute, St. Michael’s Hospital, Toronto, Ontario Canada; 8 Institute of Health Policy Management and Evaluation, University of Toronto, Toronto, Ontario Canada; 9 Department of Medicine, University of Toronto, Toronto, Ontario Canada; 10 Department of Critical Care Medicine, Cumming School of Medicine, University of Calgary, Calgary, Alberta Canada

**Keywords:** Knowledge translation, Implementation, Models, Theories, Frameworks, Diffusion, Dissemination, Research utilization

## Abstract

**Background:**

Application of knowledge translation (KT) theories, models, and frameworks (TMFs) is one method for successfully incorporating evidence into clinical care. However, there are multiple KT TMFs and little guidance on which to select. This study sought to identify and describe available full-spectrum KT TMFs to subsequently guide users.

**Methods:**

A scoping review was completed. Articles were identified through searches within electronic databases, previous reviews, grey literature, and consultation with KT experts. Search terms included combinations of KT terms and theory-related terms. Included citations had to describe full-spectrum KT TMFs that had been applied or tested. Titles/abstracts and full-text articles were screened independently by two investigators. Each KT TMF was described by its characteristics including name, context, key components, how it was used, primary target audience, levels of use, and study outcomes. Each KT TMF was also categorized into theoretical approaches as process models, determinant frameworks, classic theories, implementation theories, and evaluation frameworks. Within each category, KT TMFs were compared and contrasted to identify similarities and unique characteristics.

**Results:**

Electronic searches yielded 7160 citations. Additional citations were identified from previous reviews (*n* = 41) and bibliographies of included full-text articles (*n* = 6). Thirty-six citations describing 36 full-spectrum were identified. In 24 KT TMFs, the primary target audience was multi-level including patients/public, professionals, organizational, and financial/regulatory. The majority of the KT TMFs were used within public health, followed by research (organizational, translation, health), or in multiple contexts. Twenty-six could be used at the individual, organization, or policy levels, five at the individual/organization levels, three at the individual level only, and two at the organizational/policy level. Categorization of the KT TMFs resulted in 18 process models, eight classic theories, three determinant frameworks, three evaluation frameworks, and four that fit more than one category. There were no KT TMFs that fit the implementation theory category. Within each category, similarities and unique characteristics emerged through comparison.

**Conclusions:**

A systematic compilation of existing full-spectrum KT TMFs, categorization into different approaches, and comparison has been provided in a user-friendly way. This list provides options for users to select from when designing KT projects and interventions.

**Trial registration:**

A protocol outlining the methodology of this scoping review was developed and registered with PROSPERO (CRD42018088564).

Contributions to the literature

- There has been a proliferation of knowledge translation (KT) theories, models, and frameworks (TMFs) creating confusion for users on which to select.
- This scoping review provides a compendium and comparison of full-spectrum KT TMFs defined as those that have been used in the literature by study authors to inform their KT work and guide all four phases of knowledge translation from planning/design, implementation, evaluation, and sustainability/scalability.
- The review findings contribute to the field by providing a concise reference source for those undertaking a KT project or designing a KT intervention to assist with KT TMF selection.

## Background

There are gaps between evidence and practice. Knowledge translation (KT) has emerged as a field to bridge these knowledge-practice gaps [1, 2]. The Canadian Institutes of Health Research (CIHR) defines KT as “a dynamic and iterative process that includes the synthesis, dissemination, exchange and ethically-sound application of knowledge to improve the health of Canadians, provide more effective health services and products, and strengthen the healthcare system” [3].

The field of KT is marred by a profusion of terms. In fact, 100 terms have been found to describe knowledge translation research [4]. In the literature and among different jurisdictions, KT has been used inter-changeably with terms such as research utilization, knowledge transfer and uptake, knowledge utilization and exchange, and implementation science (IS). In particular, KT’s relationship to IS has caused confusion [5]. KT and IS are related and overlapping terms [6]. IS may be considered a sub-specialty of KT [7]. IS is defined as “the scientific study of methods to promote the systematic uptake of research findings and other evidence-based practices into routine practice to improve the quality and effectiveness of health services and care” [8]. In this paper, as with the previously published scoping review by Strifler et al., KT has been considered broadly to include implementation practice (implementing research evidence into practice) and implementation science (study of methods to promote uptake of research findings into practice) [9]. The full-spectrum KT TMFs covered in this review encompass the entire continuum of KT activities including IS. KT and IS TMFs have been referred to as KT TMFs, collectively. Further, for the practitioner, the use of these KT TMFs requires consideration of their purpose and context.

Many of KT theories, models, and frameworks, collectively referred to as “TMFs” hereafter, exist. Although four reviews have been conducted in this area to date, none of these reviews provide a comprehensive list of TMFs that include all four KT phases (planning/design, implementation, evaluation, and sustainability) [9–12]. A non-systematic review used a snowball sampling method to develop a list of 61 dissemination and implementation research TMFs [12]. A second review, which included literature from 1990 to 2014, found 41 different frameworks and models from 98 papers with a focus on research translation frameworks [11]. Further, a scoping review of published and grey literature between 2009 and 2013 identified 51 classification schemes (23 taxonomies, 15 frameworks, eight intervention lists, three models, and two other approaches) on KT interventions that could be used to integrate evidence into practice [10]. The most recently published scoping review was limited to TMFs used in cancer and chronic disease management and prevention, identifying 159 articles that met the inclusion criteria of the review [9]. Each of these studies used different research methodologies (two were narrative reviews and two were scoping reviews), literature search strategies, dates in their searches, and different inclusion/exclusion criteria. Moreover, the overlap of the KT TMFs captured in all four of these reviews was low with different TMFs included in each synthesis piece. In addition, Wensing and Grol note that the development in the fields of KT and IS has been hindered by the proliferation of TMFs for implementation, transfer, and improvement without testing, refinement, and integration [6]. It is likely that users would use, test, and or refine existing TMFs if there was a concise resource to add in their selection of a TMF. Thus, a compendium of full-spectrum KT TMFs is required. This study employs scoping review methodology to identify, describe, and compare the available full-spectrum KT TMFs.

## Methods

A protocol outlining the methodology of this scoping review was developed using the Arksey and O’Malley methods and registered with PROSPERO (CRD42018088564) [13]. The Preferred Reporting Items for Systematic Reviews and Meta-Analyses extension for Scoping Reviews (PRISMA-ScR) guidelines were followed [14].

### Search strategy

Strifler et al.’s search strategy was adapted and applied to the same databases of MEDLINE, EMBASE, and PsycINFO [9]. This required eliminating terms related to cancer and chronic diseases, limiting the search to English language and human studies, and conducting an updated search from when Strifler et al.’s search ended (i.e. from January 2016 to February 10, 2018) (Appendix A). Hand searches were conducted within KT TMF review articles published after the Strifler et al. review [11, 15], and within bibliographies of included articles. The Canadian Agency for Drugs and Technologies in Health’s (CADTH) grey matters approach, supplemented by known KT grey literature resources, was used to guide the grey literature search [16] (Appendix B). KT experts reviewed the list of KT TMFs to ensure the comprehensiveness of the final list of included studies.

### Study selection

Titles and abstracts were screened independently by two investigators (RE, SB). The inclusion criteria were:

1. English language articles.
2. Described a KT TMF as defined by Nilsen [17]. The definitions are as follows: A theory is defined as “a set of analytical principles or statements designed to structure our observation, understanding and explanation of the world.” A model is “a deliberate simplification of a phenomenon or a specific aspect of a phenomenon.” Lastly, a framework is described as “structure, overview, outline, system or plan consisting of various descriptive categories” [17]. As an entity, we considered “TMF” together as the unit when reviewing them as full spectrum.
3. Full spectrum. Specifically, a full-spectrum KT TMF is one that has been used in the literature by study authors to inform their KT work and guide all four KT phases: (i) planning/design (identifies a knowledge gap, engages stakeholders, develops an intervention), (ii) implementation, (iii) evaluation, and (iv) sustainability/scalability [9].
4. Used at any level within a system (clinical, organizational, policy).
5. Applied prospectively rather than retrospectively. A TMF that was applied only retrospectively to determine fit after project completion or for refinement was not considered. Only TMFs that were applied prospectively to support a project during its lifespan were considered.
6. Was not included in the scoping review by Strifler et al. [9].
7. Published journal articles, books, book chapters, reports, conference abstracts, and study protocols where study results (preliminary or full) had been published. Abstracts, letters, editorials, opinion articles, dissertations, and reviews were excluded.

Prior to full screening, reviewers completed a calibration exercise on 10% of retrieved articles, and inter-rater reliability was calculated for title and abstract screening using the kappa (*κ*) statistic. A moderate level of agreement (0.41 to 0.60) was considered acceptable due to the variability of this topic in the literature [18]. Titles and abstracts identified by either reviewer were included in the full-text review. Full-text articles were retrieved and reviewed in duplicate by the same two investigators (RE, SB). Reasons for exclusion of title/abstracts and full-text articles were documented using Microsoft Excel. Again, a *κ* statistic was calculated to measure agreement. Any disagreements were resolved through consensus by RE and SB. When consensus could not be reached, a third reviewer (FC and/or HH) assessed the article for eligibility.

### Data extraction items and process

Data were extracted in duplicate by two investigators independently (RE and SB). Any disagreements were resolved through consensus by RE and SB through discussion. Data elements included study characteristics (author, year, geographic region, funding source), aims of study, developmental approach, KT TMF characteristics (name, terms used to describe it-theory/model/framework), context, key components of the KT TMF, description of how it was used, primary target audience or user, levels of use, and study outcomes. As the focus of this review was identification of all full-spectrum KT TMFs, quality appraisal was not conducted nor does a tool to assess quality exist.

### Data analysis and synthesis

Data were summarized based on study characteristics and KT TMF characteristics. The KT TMFs were then categorized by two researchers (RE and JHL) according to the five categories of theoretical approaches described by Nilsen [17]. The categories include process models, determinant frameworks, classic theories, implementation theories, and evaluation frameworks (Table 1). Any disagreements were resolved through consensus by RE and JHL through discussion.

**Table 1:** Theoretical approach categories as described by Nilsen P [17]

Categories	Description
Process models	Specify steps in the process of translating research into practice.
Determinant frameworks	Classes or domains of determinants that are hypothesized or have been found to influence implementation outcomes.
Classic theories	Describe how change occurs without ambitions to actually carry out the change.
Implementation theories	Developed and adapted by researchers for potential use in implementation science to achieve enhanced understanding and explanation of certain aspects of implementation.
Evaluation frameworks	Provide a structure for evaluating implementation endeavours.

The KT TMFs within each category were described based on their primary target audience or user, context, how it has been used, and levels of use (individual, organization, or policy). Further, each KT TMF was compared and contrasted within each category with respect to its similarities and unique characteristics.

## Results

The 26 full-spectrum KT TMFs from the Strifler scoping review were assessed for inclusion within this review. Of the 26, three full-spectrum KT TMFs were excluded: one as it was action research which described a KT approach to change rather than a specific KT TMF [19], and two were applied retrospectively (the Interactive Systems Framework [20] and the Three-World View Model [21]). The remaining 23 full-spectrum KT TMFs were included [5, 22–43].

### Search results

**Fig. 1:**
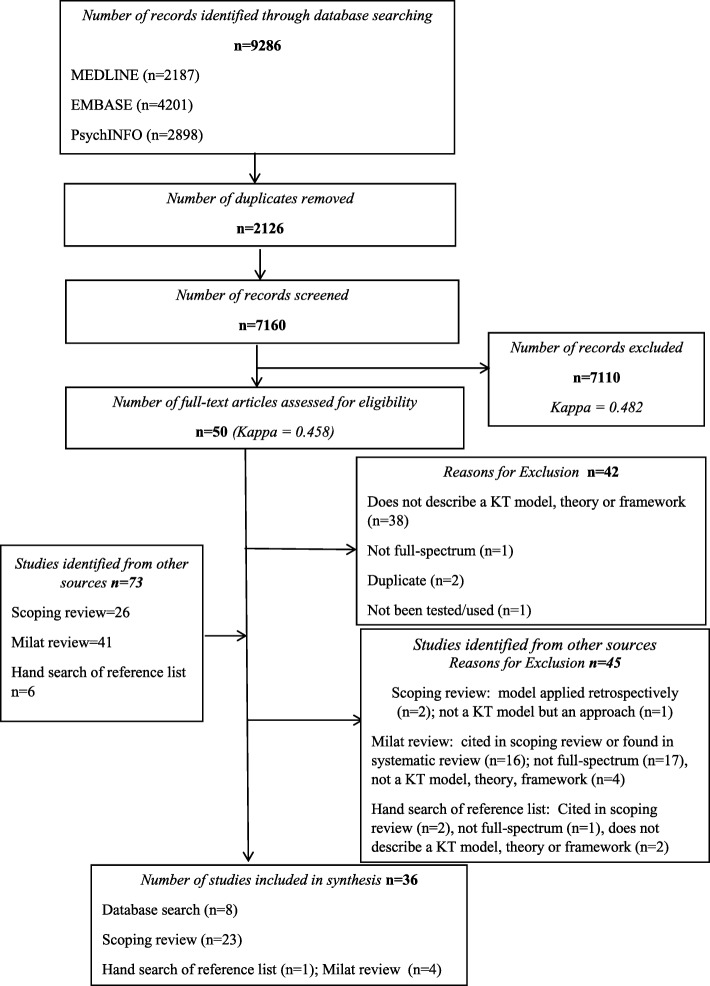
PRISMA flowchart summarizing study review and inclusion

The updated database search from January 2016 to February10, 2018, yielded 9286 articles. When duplicates were removed, 7160 unique citations were assessed (*κ* = 0.482), and 50 full-text articles were identified for further review (*κ* = 0.458) with a moderate level of agreement. After full-text review, 42 citations were excluded: 38 did not describe a KT TMF, two were duplicates, one had not been tested or used, and one KT TMF was not full-spectrum. Therefore, eight full-spectrum KT TMFs were identified [44–51]. Of the 47 references, 41 from the Milat et al. review and six articles from the hand search of the reference lists of the eight articles identified from the database search, five met the criteria for inclusion [52–56]. Eighteen were already cited in Strifler et al.’s scoping review or through the electronic database search, 18 were not full-spectrum, and six did not describe a KT TMF. The final data set included 36 full-spectrum KT TMFs (Fig. 1). Of note, there were 19 KT TMFs identified in the search that were not included in the final data set as they did not meet the criteria of full-spectrum. In particular, all 19 KT TMFs did not have the sustainability/scalability phase and two of these KT TMFs also did not have the evaluation phase (Additional file 1: Table S1).

### Description and categorization of TMFs

**Fig. 2:**
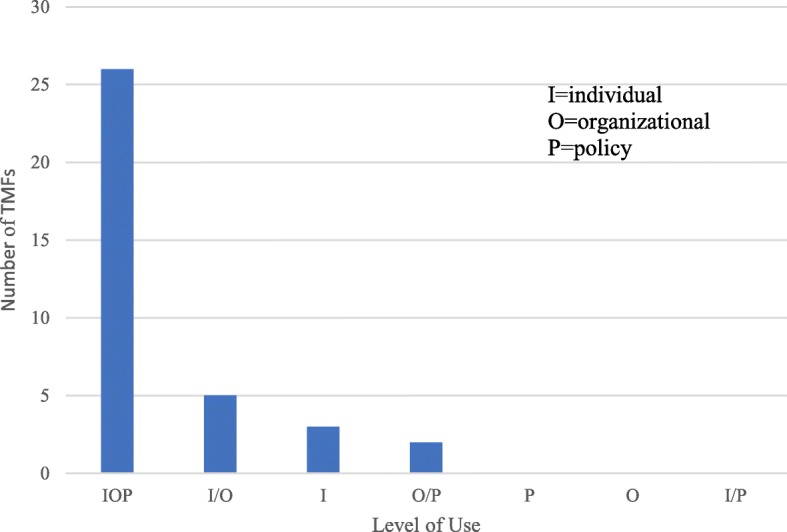
Knowledge translation theories, models, and frameworks by level of use

**Fig. 3:**
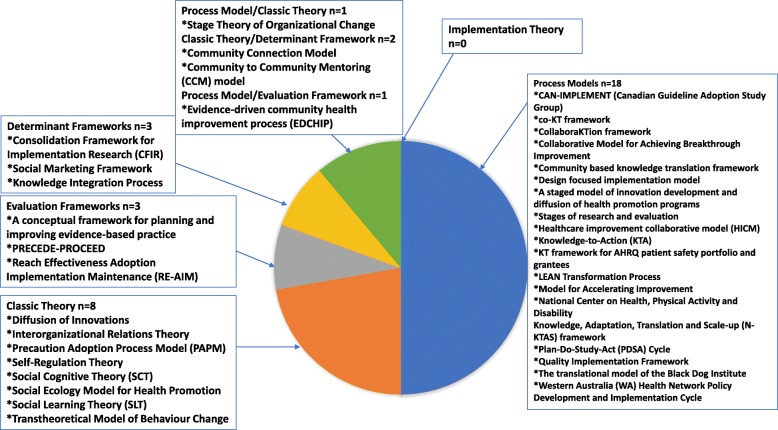
Categorization of full-spectrum knowledge translation theories, models, and frameworks (*n* = 36)

The 36 full-spectrum KT TMFs were published between 1952 and 2018, with 30 KT TMFs published since 1997 and six KT TMFs published from 1952 to 1996 [24, 27, 31, 32, 36, 37]. Twenty-three TMFs were developed in the USA [23, 25–28, 31–33, 35–39, 41, 42, 44, 48, 50, 52–54], five in Canada [5, 22, 43, 46, 56], five in Australia [24, 30, 45, 47, 51], one in India [49], and two did not have a specific geographic location [34, 55]. In 24 of the KT TMFs, the primary target audience or user was multi-level including patients/public, professionals, organizational, and financial/regulatory. The majority of the KT TMFs were used within the public health context followed by the research context (organizational, translation, health) or in multiple contexts. Twenty-six could be used at the individual, organization, or policy level (Fig. 2). Categorization of each KT TMF resulted in 18 process models [5, 22–28, 30, 45–51, 55, 56], eight classic theories [31–37, 39], three determinant frameworks [29, 40, 54], three evaluation frameworks [41, 42, 53], one that fit both the process model and classic theory categories [38], two that fit the classic theory and determinant framework categories [43, 44], and one that fit the process model and evaluation framework categories [52] (Table 2, Fig. 3). There were no full-spectrum KT TMFs that fit the implementation theory category. Additional details of each KT TMF are described in Additional files 2, 3, 4 and 5.

**Table 2:** List of full-spectrum theories, models, and frameworks by theoretical approaches categories (*n* = 36)

Categories	TMFs
Process models (*n* = 18)	CAN-IMPLEMENT (Canadian Guideline Adoption Study Group) [22] Co-KT Framework [47] CollaboraKTion Framework [46] Collaborative Model for Achieving Breakthrough Improvement [23] Community-based knowledge translation framework [56] Designed Focused Implementation Model [49] A Staged Model of Innovation Development and Diffusion of Health Promotion Programs [24] Stages of Research and Evaluation [55] Healthcare Improvement Collaborative Model (HICM) [45] Knowledge-to-Action (KTA) [5] KT Framework for AHRQ Patient Safety Portfolio and Grantees [48] LEAN Transformation Process [25] Model for Accelerating Improvement [26] National Center on Health, Physical Activity and Disability Knowledge, Adaptation, Translation and Scale-up (N-KTAS) framework [50] Plan-Do-Study-Action (PDSA) Cycle [27] Quality Implementation Framework [28] The Translational Model of the Black Dog Institute [51] Western Australia (WA) Health Network Policy Development and Implementation Cycle [30]
Classic theories (*n* = 8)	Diffusion of Innovations [31] Interorganizational Relations Theory [33] Precaution Adoption Process Model (PAPM) [35] Self-Regulation Theory [34] Social Cognitive Theory (SCT) [32] Social Ecology Model for Health Promotion [37] Social Learning Theory [36] Transtheoretical Model of Behaviour Change [39]
Determinant Frameworks (*n* = 3)	Consolidated Framework for Implementation Research (CFIR) [40] Social Marketing Framework [29] Knowledge Integration Process [54]
Evaluation frameworks (*n* = 3)	A Conceptual Framework for Planning and Improving Evidence-Based Practices [53] PRECEDE-PROCEED [42] RE-AIM [41]
Process and Classic Theory (*n* = 1)	Stage Theory of Organizational Change [38]
Classic Theory and Determinant Framework (*n* = 2)	Community Connection Model [43] Community to Community Mentoring Model (CCM) [44]
Process Model and Evaluation Framework (*n* = 1)	Evidence-Driven Community Health Improvement Process (EDCHIP) [52]

### Process models

Eighteen full-spectrum KT TMFs were process models [5, 22–28, 30, 45–51, 55, 56] (Additional file 2: Table S2). Fourteen of these had multi-level target audiences [5, 22–25, 27, 28, 30, 47, 48, 50, 51, 55, 56], whereas four had a more focussed target audience, including individual/organizational, health care, public health, or health systems [26, 45, 46, 49].

The 18 process TMFs in this category were group further based on their specific focus or similar characteristics.

### TMFs that build on existing TMFs

Twelve TMFs incorporated components of or built on existing TMFs. For instance, six TMFs have been used for quality improvement purposes and five incorporate the Plan-Do-Study-Act (PDSA) cycle [23, 25–27, 45, 49]. Another four TMFs incorporated or provided variations on the KTA framework [5]. The Knowledge-to-Action (KTA) framework was incorporated into the CAN-IMPLEMENT [22], the community-based knowledge translation model [56], the National Center on Health, Physical Activity and Disability Knowledge, Adaptation, Translation and Scale-up Framework (NCHPAD N-KTAS) [50], and the Translational Model of the Black Dog Institute [51]. In addition, the CollaboraKTion Framework [46] which focusses on population health incorporated the co-KT framework [47], making it a more iterative rather than linear process as depicted by the co-KT framework.

### TMFs that use stages to describe the KT process

Three TMFs described the KT process specifically as stages [24, 48, 55]. The Stages of Research and Evaluation are focussed on the evaluation of an intervention with six stages [55]. The Staged Model of Innovation Development’s focus is on organizational development. Its four stages are similar to the Stages of Research and Evaluation with stage 1 (basic research and design) covering the first three stages (problem definition, solution generation, intervention testing) [24]. The KT Framework for AHRQ Patient Safety Portfolio and Grantees’ focus has three stages that are similar to the other TMFs, but its focus is on moving end of grant research findings into practice [48].

### Meta-framework or network TMFs

Lastly, there were two TMFs that were distinct from the others in this category. The Quality Implementation Framework was a meta-framework of the synthesis of 25 implementation frameworks [28]. The Western Australia Health Policy Development and Implementation Cycle is a network model that incorporated the concept of a community of practice approach to build relationships between stakeholders. Both of these TMFs did not incorporate or build on other TMFs and did not consist of stages to drive process change.

Therefore, users seeking full-spectrum KT process models to provide a step by step “how to” approach to translation of knowledge into practice may wish to determine their focus and select the process KT TMF accordingly. In this category, there may be more updated versions of KT TMFs or variations of KT TMFs to contemplate and select from.

### Classic theories

Eight full-spectrum KT TMFs were found in this category [31–37, 39] (Additional file 3: Table S3). Three theories could be used for a multi-level target audience [31, 37, 39]. Three theories had a target audience at the individual level [32, 34, 36]. One provides an explanation of how change could occur with the organization or policy target audience [33], and the other was at the individual, clinic, or community level [35].

The eight theories within this category can be described as formal theories described as theories that are structured and technical and provide formal accounts of concepts [57]. Further differentiation between these theories is dependent on their purpose and level of change. For example, if the focus of the change is happening at the organizational or community levels, the Diffusion of Innovations Theory (DIT), the Interorganizational Relations Theory (IOR), or the Social Ecology Model (SE) for Health Promotion would be applicable [31, 33, 37]. If theories are required for individual health behaviour, the Transtheoretical Model (TTM) of Behaviour Change and Precaution Adoption Process Model (PAPM) would fit this purpose [35, 39]. If theories are being sought on the interpersonal environment that affect an individual’s health behaviour, the Social Cognitive Theory (SCT), Self-Regulation (SR) Theory, and Social Learning Theory (SCT) would be suitable [32, 34, 36].

DIT is a foundation theory upon which other theories are based [31]. The IOR is similar to DIT through its broad components and focus at the organizational level and SE model as it is directed at the social ecology of multiple organizations within communities [33]. Of the eight theories, the IOR is the only one that specifically provides criteria of effectiveness. The SE model is similar to DIT in terms of social systems and incorporates the notion of consequences. Its unique characteristic is the ecological analysis of health promotion [37].

The TTM and PAPM are stage theories and similar to the KT TMFs described in the process category that also specifically use stages to describe the change process [24, 48, 55]. The PAPM differs from TTM in the number of stages and its conceptualization [58]. It also considers the stage in which people may be unaware of a risk and includes a stage where people decide not to act. It does not consider commencement of risky behaviour as with TTM [35]. A notable characteristic of the TTM is that it provides ten processes of change, which can be used at each stage [39]. The TTM has been criticized as not having evidence of effectiveness as demonstrated by reviews [57, 58].

Four theories (SR, SLT, SCT, SE) are focussed on behaviour change and the environment which is key to understanding the context in which change is occurring. SR is focused on self-regulation or taking a response or behaviour and replacing it with a less common, but more desired response [34]. It considers the environment like the SE model and IOR in shaping behaviour [33, 37]. Its second dimension of self as it relates to others is similar to SLT and SCT [32, 36]. SLT is about learning and meaning. SCT is based on SLT and bridges the gap between learning and behaviour [36]. SCT also adds the notion of self-efficacy and self-regulation like SR [32].

Therefore, users seeking full-spectrum KT theories, to explain how change occurs need to consider these factors when selecting from these eight theories. As Davidoff et al. describe, it is not about using theory but about making explicit the particular theory, that is actually used [57]. More importantly, it may not just be a selection of one theory but a blending of theories that may be required [57, 58].

### Determinant frameworks

Three full-spectrum KT TMFs were found in this category [29, 40, 54] (Additional file 4: Table S4). All three had multi-level target audiences.

All three frameworks provide determinants that could influence change and implementation. The Social Marketing Framework uses the principles, techniques, and tools of marketing [29]. It overlaps with CIFR on the identification of target audience, internal factors (degree of readiness), external factors (self-efficacy), and perceived risk. As with CIFR, it also integrates both qualitative and quantitative research methods. In addition, the Social Marketing Framework considers price in terms of incentives and disincentives, whereas CIFR considers costs in terms of the intervention. However, the Social Marketing Framework also has distinguishing features such as how the five Ps are inter-related and additional determinants such as audience segmentation, competition, and exchange [29].

The Knowledge Integration Process illustrates a cycle of five overlapping phases from problem identification and discovery to population health outcomes [54]. Each phase consists of determinants to consider, but it does not go into detail on the determinants. Similar to CIFR and the Social Marketing Framework, the Knowledge Integration Process considers stakeholder engagement.

Lastly, CFIR is meta-theoretical framework based on existing theories and empirical studies. It provides tools and a guide that can be used alongside the framework. CIFR can be used to assess barriers and facilitators for behaviour change. However, one drawback is that it does not depict interrelationships between these determinants [40].

Therefore, users seeking full-spectrum KT determinant frameworks to identify barriers and facilitators that may influence implementation outcomes have three distinct frameworks to choose from. Moreover, there are other non-full-spectrum KT determinant frameworks that may be useful to consider in addition to these three [59].

### Evaluation frameworks

Three full-spectrum KT TMFs fit this category [41, 42, 53] (Additional file 4: Table S4). Two had multi-level [42, 53] and one had individual or organizational levels [41] as their primary target audience.

All three TMFs can be used to evaluate interventions and the extent to which the interventions are applied in practice. Reach, Effectiveness, Adoption, Implementation, and Maintenance (RE-AIM) focusses on five elements and is the only full-spectrum evaluation framework that is solely quantitative in nature [41]. This framework is unique as it allows for the assessment of intervention impact over time and for comparison of interventions. However, RE-AIM assumes that the five elements are equal, which may not be the case. In addition, the time interval for assessing implementation (6 months to a year) and maintenance (two or more years), respectively, is arbitrary [41].

The Conceptual Framework for Planning and Improving Evidence-based Practices is focussed on planning and improving best practices. Its public health impact component is adapted from RE-AIM. In contrast to RE-AIM, it provides a series of questions for the five elements. However, not all the questions are comprehensive and some may not be able to be used for all practices [53]. Further, it does not provide practical guidance on what to do with the information obtained.

Similar to the Conceptual Framework, PRECEDE-PROCEED also focusses on the adaptation of best practices. It provides a generic framework and logic model to assess the linkage between activities and long-term impacts [42]. In contrast to the Conceptual Framework, this framework describes phases to assist with generating information for subsequent decisions rather than posing questions that need to be addressed. Of the three evaluation frameworks, it is the only one that provides linkage between evaluation being part of implementation, monitoring, and continuous quality improvement.

Therefore, users seeking full-spectrum KT evaluation frameworks to evaluate interventions may need to consider these factors when selecting from these three frameworks.

### KT theories, models, and frameworks that fit more than one theoretical approach category

Four KT TMFs fit more than one theoretical approach category [38, 43, 44, 52] (Additional file 5: Table S5). Two had multi-level [43, 44], one had organizational level [38], and one had community [52] as their primary target audience.

In comparison to the other process category TMFs, the Stage Theory of Organizational Change is similar to the Staged Model of Innovation Development and the Stages of Research Evaluation [55], as it provides stages for moving research into practice and consists of four distinct stages. In relation to the classic theory category, it is similar to the DIT [31] and IOR [33], with its focus on how change occurs at the organizational level.

The Community Connection Model is not based on specific behavioural theories rather the author’s reflection on the development and implementation of a chronic disease program. However, it does provide a broad overview of the components that necessitate change to occur similar to other theories. Its four components can also be considered determinants for change to occur. Moreover, one component is the alignment of policy to facilitate building partnerships, which is similar to the Social Marketing Framework that considers other providers and community organizations as a determinant of change.

In relation to the classic theories, the Community to Community Mentoring Model (CCM) is based on elements from DIT and the SCT TMFs including innovation characteristics, peer modeling to influence behaviour, and self-efficacy. CCM also incorporates community-based participatory research (CBPR) principles. It overlaps with the Community Connection Model through the partnership component. Moreover, it also provides three additional components of context, community-based participatory research principles, and innovation elements that are also similar to the determinants within CIFR.

The Evidence-driven Community Health Improvement Process (EDCHIP) builds on CHIP and  incorporates the search and organization of scientific literature and RE-AIM, respectively. EDCHIP is similar to the KTA process TMF [5] through the synthesis of scientific knowledge (the funnel) and specific implementation steps (the action cycle). Further, it replicates RE-AIM [41] within its analysis and implementation cycle. It is novel in that it incorporates elements from two distinct full-text KT TMFs from the process and evaluation categories and integrates them for a more comprehensive TMF.

Thus, these four cross-cutting KT TMFs offer yet other options and may be suitable for users seeking more than one theoretical approach in a KT TMF.

## Discussion

### Key findings

Three key findings emerged from this scoping review: an up-to-date compilation of full-spectrum KT TMFs using scoping review methodology; categorization of these KT TMFs based on five categories of approaches; a detailed description of each KT TMF; and comparison within each category to assist with their selection for a KT project or intervention.

This scoping review identified 36 full-spectrum KT TMFs. The use of full-spectrum KT TMFs to anchor a KT project is considered an important and foundational starting point. Rather than the development of yet another KT TMF, users are encouraged to select from this current list and then share their experiences on application (Additional files 2, 3, 4, and 5). This approach will help to further develop and improve the theoretical foundation that KT science is built on.

This review found that there are full-spectrum KT TMFs that fit all but one of the five theoretical categories. Although Nilsen emphasized that these categories can overlap, it is important to recognize the differences between them so that users can select the KT TMF most appropriate for the situation [17]. Indeed, there were four KT TMFs that fit more than one theoretical approach category [38, 43, 44, 52]. Furthermore, half of the full-spectrum KT TMFs fall into the process category. Over the past decade, a major focus within the field of KT has been on developing steps and processes that support the application of KT TMFs [17].

The lack of a full-spectrum KT TMF within the implementation theory category may reflect that these KT TMFs are used in implementation science to understand and explain aspects of implementation. Thus, they may not cover all four phases of KT, but instead focus on the planning/design and implementation phases. An example is the COM-B (Capability, Opportunity, Motivation and Behaviour) [60]. This framework posits that the presence of COM can influence behaviour which in turn influences COM. It has been used to explain why and how implementation occurs.

Comparison of the KT TMFs within each category highlighted similarities and unique characteristics. Within the process category, two thirds of the KT TMFs incorporated other TMFs or provided variations of previous TMFs. Six of these TMFs had a focus toward quality improvement. The classic theory category presented TMFs that were foundational, such as the DIT. The theories within this category concentrated on the level of change at the organizational, community, or behavioural change related to self. Both determinant and evaluation framework categories had three TMFs each. The determinant category offered a meta-theoretical framework, with two simpler determinant frameworks. The evaluation category offered three very distinct TMFs, with one quantitative in nature. The final category afforded TMFs that could fit more than one category. As such, they would be useful for users seeking more than one theoretical approach.

This list of 36 full-spectrum KT TMFs and their categorization provides a starting point for any KT project or intervention. By providing a detailed description and comparison of TMFs within each category, users can scan the available TMF options and determine which may be best suited to their KT approach. For example, a user searching for a *process* KT TMF to apply within multiple target audiences at the *policy level* could select from three potential process TMFs, (KTA, Collaborative Model for Achieving Breakthrough Improvement, PDSA cycle) [5, 23, 27]. Moreover, if users are looking for a KT TMF that has both a process and evaluation approach, they could select the EDCHIP model [52]. Therefore, users have a concise list of KT TMFs to select from based on approach, purpose, context, and the level of use [61].

Birken et al. has suggested a more complex process for selecting a KT TMF that involves 19 criteria [62]. However, not dissimilar to how the KT TMFs were described in this review, the criteria ranked most important were empirical support, application to a specific setting or population, explanatory power or testability, description of a change process, and analytical level (macro-policy level, meso-health care organization level, and micro-clinical level). Furthermore, the authors have developed the T-CAST: an implementation theory comparison and selection tool with 16 specific criteria, organized within four categories, applicability, usability, testability, and acceptability. Users of the T-CAST tool will find it helpful for deciding whether a specific TMF is relevant for their project or for deciding which of several TMFs is most relevant for their project. As such, the T-CAST aids in the selection of TMFs from among a candidate list [63]. Thus, this scoping review provides a list for users to review the range of candidate full-spectrum TMFs available and subsequently apply the T-CAST tool to compare and select a KT TMF.

Another consideration in selection of a KT TMF is the application of objective criteria on ease of use or usability. Although not a focus of this review, the usability of each KT TMF would provide an added dimension for users to consider when selecting KT TMFs. Usability has been defined as “the quality or state of being usable: ease of use” [64]. The T-CAST tool includes usability criteria for practitioners and researchers. Each criterion is scored as 0 for poor fit, 1 for moderate fit, and 2 for good fit. This scoring system could be used to objectively identify usability from a user perspective and assist in KT TMF selection [63]. Alternatively, drawing from the computer system literature, the System Usability Scale (SUS) could provide another way to objectively rank each KT TMF for usability [65]. The application of usability criteria to KT TMFs that are full-spectrum is an area that could be considered for future research.

Therefore, this scoping review can be used as a resource and starting point from which users can select a full-spectrum KT TMF. The T-CAST tool or a usability scale could then be applied by users to compare among these KT TMFs to assist with potential fit of the KT TMF or TMFs used in combination for their project.

### Strengths

This scoping review followed a rigorous protocol, building on a previous high-quality scoping review [9] in a unique manner that enabled the capture of full-spectrum KT TMFs. Moreover, whereas the previous scoping review [9] focussed on cancer and chronic disease, this scoping review modified the search strategy to identify KT TMFs regardless of context and provided details and comparison of each KT TMF. The comprehensive nature of the search included a search of the grey literature, examination of review articles published after the scoping review, and a review of the final list by KT experts, which helped minimize the risk of missing a relevant full-spectrum KT TMF. To our knowledge, it is the only scoping review that provides a compendium and comprehensive description of full-spectrum KT TMFs. The scoping review also compares each KT TMF within categories to allow users to better understand and assist with their selection.

### Limitations

Articles published in languages other than English were not incorporated into the search strategy due to lack of resources. So, the review may be subject to language bias. In addition, there may have been misclassification of TMFs during the search. However, the BeHEMOth search strategy utilizes different terms for theory, model, and framework to minimize the risk of misclassification. Kappa agreement between the reviewers was moderate and could be attributed to the lack of clarity in the literature of how a KT TMF is defined.

Nilsen’s definitions of a theory, model, and framework were used, but there are other definitions that could have been used to define these terms [66]. Moreover, often these three terms are used inter-changeably in the literature and cause further confusion. These terms are not synonyms and there is a need for future research to provide guidance on how to determine which approach to use, i.e. a theory, model or framework based on the research study and context prior to the actual selection of the TMF.

Categorization of TMFs was based on the definitions provided by Nilsen. This work is meant to be a guideline, and the distinctions among the categories are not precise. Hence, misclassification could have occurred due to interpretation. However, two reviewers conducted this categorization in duplicate to ensure reliability and any disagreements were resolved through dialogue. The use of Nilsen’s taxonomy can help as a starting point with selecting which full-spectrum KT TMF to use based on its purpose [17]. For example, if the purpose is to employ a change in practice, a process model such as KTA could be used [5]. Alternatively, if it is to understand or explain how a change occurs, the DIT could be selected [31]. This scoping review has provided a reference and categorization so that users are not left to sift through the numerous KT TMFs. Moreover, further refinement of Nilsen’s categories and categorization of commonly used KT TMFs that are not full-spectrum would be a beneficial piece for future research.

Providing a compendium of full-spectrum TMFs only may miss several commonly used TMFs such as those cited by Birken et al. [62]. In this study, CIFR, RE-AIM, and DIT have been cited as the top three most commonly used TMFs, and they also were classified as full-spectrum in this scoping review. However, other commonly used TMFs such as Promoting Action on Research Implementation in Health Services (PARIHS) Framework [67] and Normalization Process Theory [68] did not make the full-spectrum list. PARIHS and Normalization Process Theory were cited by Strifler et al.’s scoping review. However, they were not classified as full-spectrum. PARIHS did not have the planning/design and sustainability/scalability phases and the Normalization Process Theory was missing the implementation and sustainability phases [9]. Nonetheless, there is added value in presenting KT TMFs that are full-spectrum as they meet the criteria for all four KT phases considered important for users when implementing KT projects or interventions. In addition, users may want to explore the use of both full-spectrum KT TMFs and other commonly used KT TMFs. Combining KT TMFs for implementation has been shown to be beneficial given the context and complex nature of implementation science itself [69].

The full-spectrum definition of KT stages is based on the KTA framework [5], but other KT TMFs could have been used as a basis for defining full-spectrum. In addition, there may be different perspectives in the classification of a KT TMFs as full-spectrum. For example, although the RE-AIM framework was primarily designed to be used as an evaluation framework focussed only on the evaluation phase of the KT process, its use has evolved and is now used in planning, assessing progress and reporting results [70]. Moreover, given that the Strifler et al. scoping review did include it as full-spectrum [9], it was also included in this scoping review. Lastly, although time and resources were invested to obtain the original source of publication of the actual KT TMF, it was well worth the effort.

## Conclusions

This scoping review provides a summary of the full-spectrum KT TMFs that could be used as a foundation for clinicians, researchers, and policy makers, undertaking KT projects within the health care context. The application of an existing KT TMF is recommended for all applied KT projects and interventions [9]. It is only by further understanding, utilizing, and evaluating these KT TMFs that the field of KT science can advance and ultimately improve the implementation of evidence into practice.

### Supplementary information

**Additional file 1: Table S1.** List of excluded theories, models, and frameworks that did not meet full-spectrum KT phase(s) (*n* = 19)

**Additional file 2: Table S2.** Full-spectrum KT theories, models, and frameworks that fit the Process Models Category (*n* = 18)

**Additional file 3: Table S3.** Full-spectrum KT theories, models, and frameworks that fit the Classic Theories Category (*n* = 8)

**Additional file 4: Table S4.** Full-spectrum KT theories, models, and frameworks that fit the Determinant or Evaluation Frameworks Category (*n* = 6)

**Additional file 5: Table S5.** Full-spectrum KT theories, models, and frameworks that fit More than one Theoretical Approach Category (*n* = 4)

## Data Availability

Not applicable

## Appendix A

### Ovid MEDLINE Search Strategy Adapted from Strifler et al 2018

Database: Ovid MEDLINE(R) In-Process & Other Non-Indexed Citations and Ovid MEDLINE(R) <1946 to Present>

--------------------------------------------------------------------------------

Database: Ovid MEDLINE(R) In-Process & Other Non-Indexed Citations and Ovid MEDLINE(R) <1946 to Present>

--------------------------------------------------------------------------------

1 (knowledge adj2 (application or broke$ or creation or diffus$ or disseminat$ or exchang$ or implement$ or management or mobili$ or translat$ or transfer$ or uptak$ or utili$)).tw.

2 (evidence$ adj2 (exchang$ or translat$ or transfer$ or diffus$ or disseminat$ or exchang$ or implement$ or management or mobil$ or uptak$ or utili$)).tw.

3 (KT adj2 (application or broke$ or diffus$ or disseminat$ or decision$ or exchang$ or implement$ or intervent$ or mobili$ or plan$ or policy or policies or strateg$ or translat$ or transfer$ or uptak$ or utili$)).tw.

4 (research$ adj2 (diffus$ or disseminat$ or exchang$ or transfer$ or translation$ or application or implement$ or mobil$ or transfer$ or uptak$ or utili$)).tw.

5 ("research findings into action" or "research to action" or "research into action" or "evidence to action" or "evidence to practice" or "evidence into practice").tw.

6 Translational Medical Research/

7 Diffusion of Innovation/or (diffusion adj2 innovation).tw.

8 (("systematic review$" or "knowledge synthes$") adj5 ("decision mak$" or "policy mak$" or "policy decision?" or "health polic$")).tw.

9 (("systematic review$" or "knowledge synthes$") adj2 (application or implement$ or utili?ation or utilize? or utilise? or utili?ing)).tw.

10 research utili?ation.tw.

11 ((evidence base$ or evidence inform$) adj2 (decision$ or plan$ or policy or policies or practice or action$)).tw.

12 or/1-11

13 (model* or theor* or concept* or framework*).tw.

14 12 and 13

15 limit 14 to english

16 limit 15 to yr = 2016-current

## Appendix B

### Grey literature websites

In addition to websites listed in the CADTH grey matter approach, the following websites were searched:

KT Canada Clearinghouse (http://ktclearinghouse.ca/tools/uncategorized)

Implementation Central (http://www.implementationcentral.com/index.html)

Alberta Innovates Health solutions https://spor.albertainnovates.ca/the-alberta-spor-support-unit/knowledge-translation-platform/

Consolidated Framework for Implementation Research (CFIR) (http://cfirguide.org/)

National Collaborating Center for Methods and Tools (NCCMT) (http://www.nccmt.ca/resources/registry)

Cochrane Community (http://community.cochrane.org/review-production/knowledge-translation/framework-and-implementation-plan)

## Abbreviations

AHRQ: Agency for Healthcare Research and Quality
BeHEMOth: Behaviour of interest; Health context; Exclusions; Models or Theories
CADTH: Canadian Agency for Drugs and Technologies in Health
CBPR: Community-based participatory research
CDC-OPS: Center for Disease Control and Prevention’s Obesity Prevention Strategy
CCM: Community to community mentoring model
CFIR: Consolidated Framework for Implementation Research
CHIP: Community Health Improvement Process
CIHR: Canadian Institutes of Health Research
COM-B: Capability, Opportunity, Motivation and Behaviour
DIT: Diffusion of Innovations Theory
EBI: Evidence-based interventions
EDCHIP: Evidence-Driven Community Health Improvement Process
HCIM: Healthcare Improvement Collaborative Model
IOR: Interorganizational Relations Theory
KT: Knowledge translation
KTA: Knowledge-to-Action
NCCMT: National Collaborating Center for Methods and Tools
NCHPAD N-KTAS: National Center on Health, Physical Activity and Disability Knowledge, Adaptation, Translation and Scale-up Framework
PAPM: Precaution Adoption Process Model
PARIHS: Promoting Action on Research Implementation in Health Services
PEFL: Policy effectiveness feasibility loop
PDSA: Plan-Do-Study-Act
PRECEDE-PROCEED: Predisposing, Reinforcing and Enabling Constructs in Educational Diagnosis and Evaluation-Policy, Regulatory, and Organizational Constructs in Educational and Environmental Development
PRISMA-ScR: Preferred Reporting Items for Systematic Reviews and Meta-Analyses extension for Scoping Reviews
RE-AIM: Reach, Effectiveness, Adoption, Implementation, and Maintenance
SE: Social Ecology Model of Health
SCT: Social Cognitive Theory
SLT: Social Learning Theory
SR: Self-Regulation Theory
TMF: Theory, model, and framework
TTM: Transtheoretical Model
T-CAST: Implementation theory comparison and selection tool
SONAR: Social Networking App/Action for Resilience
SUS: System Usability Scale
USA: United States of America
VHA: Veterans Health Administration
WA: Western Australia
