# Supplementary material for: A scoping review of full-spectrum knowledge translation theories, models, and frameworks
Source: Implement Sci. 2020 Feb 14;15:11. doi: 10.1186/s13012-020-0964-5 (PMC7023795; doi:10.1186/s13012-020-0964-5)
Supplement: Supplementary file 1 — Additional file 1: Table S1. List of excluded theories, models, and frameworks that did not meet full-spectrum KT phase(s) (n = 19) [file 13012_2020_964_MOESM1_ESM.docx]

Additional file 1: Table S1 List of excluded theories, models, and frameworks that did not meet full-spectrum KT phase(s) (*n* = 19)

| Theory, model, framework | Reference | Stages of KT—planning/design | Stages of KT—implementation | Stages of KT—evaluation | Stages of KT—sustainability/scalability |
| --- | --- | --- | --- | --- | --- |
| From electronic searches (*n* = 1) | | | | | |
| A knowledge translation framework on ageing and health | Ellen 2017 |  |  |  | x |
| From hand search of reference list (*n* = 1) | | | | | |
| Evidence Integration Triangle | Glasgow 2012 |  |  |  | x |
| From Milat review (*n* = 17) | | | | | |
| Evidence-based public health (EBPH) models | Brownson 2009 |  |  |  | x |
| Implementation Science | Sivaram 2014 |  |  |  | x |
| Overarching knowledge translation framework | Colquhoun 2014 |  |  |  | x |
| Policy effectiveness-feasibility loop (PEFL) | Bowman 2012 |  |  |  | x |
| LEAD framework | Kumanyika 2012 |  |  |  | x |
| Policy-into-practice intervention for management of low back pain | Slater 2012 |  |  |  | x |
| Research-Practice Integration framework | Vivian 2012 |  |  |  | x |
| Evidence-informed decision making | Ward 2011 |  |  | x | x |
| Translation framework for public health | Ogilvie 2009 |  |  |  | x |
| Translational research paradigm | Spoth 2008 |  |  | x | x |
| Model for closing the evidence-to practice gap | Lang 2007 |  |  |  | x |
| Knowledge integration model | Gauthier 2005 |  |  |  | x |
| Evidence-informed policy and practice | Bowen and Zwi 2005 |  |  |  | x |
| Framework for transforming knowledge into practice | Neufeldt 2004 |  |  |  | x |
| Contextual knowledge translation framework | Ho 2004 |  |  |  | x |
| Program assessment feedback model | Sneden 2006 |  |  |  | x |
| International Obesity Task Force Evidence requirements for obesity prevention | Swinburn 2005 |  |  |  | x |
