# Supplementary material for: A scoping review of full-spectrum knowledge translation theories, models, and frameworks
Source: Implement Sci. 2020 Feb 14;15:11. doi: 10.1186/s13012-020-0964-5 (PMC7023795; doi:10.1186/s13012-020-0964-5)
Supplement: Supplementary file 2 — Additional file 2: Table S2. Full-spectrum KT theories, models, and frameworks that fit the Process Models Category (n = 18) [file 13012_2020_964_MOESM2_ESM.docx]

Additional file 2: Table S2 Full-spectrum KT theories, models, and frameworks that fit the Process Models Category (*n* = 18)

| Name of KT theory, model, or framework, author, source, year | Description | Primary target audience or user | Context | How has it been used? | Level of use (I, O, P) |
| --- | --- | --- | --- | --- | --- |
| CAN-IMPLEMENT (Canadian Guideline Adoption Study Group), Harrison, Implementation Science, 2013 [22] | Knowledge to Action (KTA) Framework (Graham et al, 2006) (5) with integration of guideline adaptation; Guideline adaptation is embedded in the  Knowledge to Action cycle as part of a new three phase model:  - PHASE 1 Identification and Clarification of Practice Issue/Problem  - PHASE 2 Solution Building  - PHASE 3 Implementation, Evaluation and Sustainability | Multi-level | Cancer guideline implementation | Five cases were purposefully sampled from self-identified groups and followed as they used a structured method and resources for guideline adaptation. Cases received the ADAPTE Collaboration toolkit, facilitation, methodological and logistical support, resources and assistance as required. | IOP |
| Integrated KT Framework, Kitson A, Implementation Science, 2013 [47] | The development of the integrated KT Framework (called the co-creating or co-KT Framework). The four areas are as follows: 1. There is a need for explicit, theory informed approaches to KT within population health studies. Formalized collaborative approaches need to be evident within any KT framework at each step of the research process; 3. The roles of stakeholders and researchers need to be negotiated, structured and formalized; and 4. KT frameworks need to explicitly describe what counts as evidence, how it is developed, and by whom. Five steps in study and researcher context: step 1 initial contact and framing the issue, step 2: refining and testing, step 3: interpreting, contextualizing and adapting the knowledge base, step 4: implementing and evaluating; step 5 embedding and translating. | Multi-level (policy and community) | Port Lincoln, Australia, chronic disease | It was applied to LINKIN study: management of musculoskeletal problems and the management of pain associated with chronic conditions. | IOP |
| CollaboraKTion Framework, Jenkins EK, Health Research Policy and Systems, 2016 [46] | Based on the co-KT framework. Five components: contacting and connection, deepening and understandings, adapting and applying the knowledge base, supporting and evaluating continued action, transitioning and embedding-based on co- KT framework from Kitson et al. Kitson is linear process. It is an iterative and a non-linear process. | Community-based organizations, public health, disease prevention, health of communities/populations | Rural town in BC Canada | Requires ongoing empirical testing but the Kitson co-KT model was applied to the SONAR project. Used through adaptation of original framework in practice-SONAR. | IOP |
| Collaborative Model for Achieving Breakthrough Improvement/Break-through series model, Institute for Healthcare Improvement white paper; model developed in 1995, paper published 2003 [23] | Breakthrough series model; Topic Selection, Faculty Recruitment, Enrollment of Participating Organizations and Teams, Learning Sessions, Action Periods, The Model for Improvement, Summative Congresses and Publications, Measurement and Evaluation. | Multi-level | Multiple settings, health care improvement settings for multiple contexts | Multiple application. But model has been used extensively. 891 teams involved up to 2001. Model has been used in Quantum Leaps in Patient Safety, Quality Assurance Project—Russia, Health Disparities Collaboratives to eliminate health disparities for 12 million underserved Americans The Veterans Health Administration (VHA). | IOP |
| Community-based knowledge translation framework, Campbell B, Journal of continuing education in the health professions, 2010 [56] | The Ottawa Model of Research Use (OMRU), a knowledge translation framework, was used to guide the translation of that generative knowledge into action, and the more current knowledge-to-action (KTA) (5) conceptual framework provided the rationale for the graphical depiction of engagement of a rural community in knowledge translation. Five phases and five stages. | Multi-level | Community research | Child health in a rural community. | IOP |
| Design Focused Implementation Model, Ramaswamy R, Int J Ment Health System, 2018 [49] | Development of a framework for implementation in low resource context-informed by review of existing frameworks. Three interlinked phases: design, implementation and improvement; evaluation is woven into each of these with appropriate evaluation questions for each component of the model. Phases used other models, i.e. design phase: local theory of change, mental healthcare plan, service delivery process maps; implementation phase used interactive system framework; improvement phase: customized version of lean six sigma; evaluation used principles of learning evaluation. Iterative improvements of the initiative (PDSA cycle and QI) monitoring system to manage/sustain the change. | Low and middle-income countries delivery of health systems; low income/resource community/organization | Three levels of health system (community, primary care and district hospitals); mental health disorders | The model was then applied to implement a program for treatment of three mental disorders (depression, alcohol use disorder and psychosis) (a case study). | IOP |
| A Staged Model of Innovation Development and Diffusion of Health Promotion Programs/A Staged Approach to Innovation Development and Diffusion/Diffusion of Health Promotion Innovations, Oldenburg B, Health Promotion Journal of Australia, 1996 [24] | This framework represents an adaptation of the more classical program development models along with other models that has been used to characterize organizational development and change. Stage 1: Basic research and program development; Stage 2: Innovation development; Stage 3: Innovation diffusion; Stage 4: Institutionalization and sustainability. | Multi-level | Public health, health promotion innovation research and implementation | Applied retrospectively to 12 journals, case studies described, applied to Fresh start program | IOP |
| Stages of Research and Evaluation, Nutbeam D Evaluation in a Nutshell 2006 book [55] | 1. Problem definition; 2. Solution generation; 3. Intervention testing; 4. Intervention demonstration; 5. Intervention dissemination; 6. Program monitoring; Stages 1 and 2 indicate the importance of a range of different forms of descriptive research in the development of an intervention plan, as well as formative evaluation of the development of program components. Stage 3 intervention testing represents the evaluation of the effectiveness of an individual program. This included process evaluation and assessment of impact and outcome of the individual program (outcome evaluation). On the basis of evidence produced at this stage, decisions can be made on whether its use and adoption should become more widespread. Stage 4 the intervention is replicated and tested in other settings to assess whether or not it works as well in other populations or places. Stage 5, the research focus is on the dissemination process and on maximizing the reach of the intervention, thereby maximizing the potential public health benefit. This requires a focus on understanding the processes of implementation in different settings, and a decreasing focus on assessing the magnitude of the impact on outcomes. Stage 6-when a program is widely adopted, it is the phase of program sustainability and maintenance (or program monitoring). This phase highlights the importance of continued monitoring of quality processes but distinguishes between this more routine process and the more formal research and evaluation methods that are required in the preceding stages. | Multi-level | Public health, health promotion, research and industry | Youth smoking | IOP |
| Healthcare Improvement Collaborative Model (HICM), Edward K, International Journal for Quality in Healthcare, 2017 [45] | The model was developed using the conceptual framework of models put forward through the IHI Breakthrough College Series and the Johns Hopkins quality and safety research group’s Translating Evidence into Practice (TRiP) model. This is a hybrid model of two models that resulted in the healthcare improvement collaborative model. The model comprises of frontline staff engagement and local topic selection. These two points of difference to the IHI model are important since staff generate their own priorities (topic selection) and can focus attention to specific improvement foci that is particular to the site. Teams are engaged in both formal and informal environments. The HICM consists of topic selection, recruitment of expert advisors, connection period, action period and team support. | healthcare system | Multi-site, multi-state, multi-sector organization | The HICM was developed and piloted from 2012 to 2016 for the focused topic of the management of inadvertent perioperative hypothermia across nine hospitals within Australia. | IOP |
| Knowledge-to-Action (KTA), Graham I, Journal of Continuing Education in Health Professions, 2006 [5] | For conceptual and illustrative purposes, KTA process is divided into two concepts: knowledge creation and action, with each concept comprised of ideal phases or categories. The action phases may occur sequentially or simultaneously, and the knowledge phases may influence the action phases. The funnel symbolizes knowledge creation, and the cycle represents the activities and processes related to use or application of knowledge (action). | Multi-level | Continuing education in health professional | Multiple applications | IOP |
| KT Framework for AHRQ Patient Safety Portfolio and grantees, Nieva VF, Advances in Patient Safety, 2005 [48] | Developed the conceptual framework to assist in the dissemination of research findings among grantees and, more importantly, among point-of-care providers who directly influence the quality of patient care. The broad objective of this framework is to maximize and accelerate the utilization of results emerging from AHRQ’s patient safety portfolio of grants and contracts. Developed a framework that synthesizes concepts from various related literatures of knowledge transfer, social marketing, social and organizational innovation, and behaviour change. There are three major knowledge transfer stages: knowledge creation and distillation, diffusion and dissemination, and organizational adoption and implementation of innovations and institutionalization-process actors and activities. | Multi-level-administrators and managers, and health care professionals, such as physicians, nurses, pharmacists and laboratory technicians | Patient Safety portfolio and patient safety grantees; AHRQ USA | Facilitation of Evidence-Based Nursing Practice During Military Operations. | IOP |
| LEAN Transformation Process, Lean Enterprise Institute, 2011, website: <http://www.lean.org/whatslean/principles.cfm> [25] | The five-step thought process for guiding the implementation of lean techniques is easy to remember, but not always easy to achieve:  1. Specify value from the standpoint of the end customer by product family.  2. Identify all the steps in the value stream for each product family, eliminating whenever possible those steps that do not create value.  3. Make the value-creating steps occur in tight sequence so the product will flow smoothly toward the customer.  4. As flow is introduced, let customers pull value from the next upstream activity.  5. As value is specified, value streams are identified, wasted steps are removed, and flow and pull are introduced, begin the process again and continue it until a state of perfection is reached in which perfect value is created with no waste. | Multi-level | Automotive industry | Multiple applications | IOP |
| Model for Accelerating Improvement Associates in Process Improvement Langley, 2009 website; <http://www.ihi.org/resources/Pages/HowtoImprove/default.aspx>  Langley, the improvement guide: a practical approach to enhancing organizational performance, 2 edition 2009 [26] | Model for Improvement asks three questions: what are we trying to accomplish, how will we know that a change is an improvement, what change can we make that will result in an improvement; changes are then put through the PDSA cycle. | Clinical or organizational | Health care organizations | Multiple applications | IO |
| National Center on Health, Physical Activity and Disability (NCHPAD)  Knowledge, Adaptation, Translation and Scale-up (N-KTAS) framework  Rimmer JH, Journal of Neurologic Physical Therapy, 2016 [50] | Integrates the work it has been doing in knowledge creation and dissemination with the growing need to advance work in implementation facilitation. KTA framework by Graham et al (5) is integrated in N-KTAS. Four sequencing strategies: strategy 1—new evidence—and practice-based knowledge is collected and adapted for the local context (i.e. community); strategy 2—customized resources are effectively disseminated to key stakeholders including rehabilitation professionals with appropriate training tools; strategy 3—NCHPAD staff serve as facilitators assisting key stakeholders in implementing recommendations; strategy 4—successful elements of practice (e.g. guideline, recommendation, adaptation) are archived and scaled to other rehabilitation providers. In the aggregate, these 4 strategies are aimed at promoting Community Health Inclusion, which is defined as the opportunity for people with disabilities to have equal access to the same health/wellness initiatives offered to other members of the community. | Multi-level: community-at-large, health care facilities, community-based institutions/organizations, schools and workplace | People with disabilities/rehab, health implementation focus | CDC example worked through theoretically. | IOP |
| Plan-Do-Study-Act (PDSA) Cycles, Deming WE, website: <https://deming.org/explore/p-d-s-a> 2018; Mowan/Clifford Norman pub 1950; PDSA created from 1986 to 1993 [27] | The cycle begins with the Plan step. This involves identifying a goal or purpose, formulating a theory, defining success metrics and putting a plan into action. These activities are followed by the Do step, in which the components of the plan are implemented, such as making a product. Next comes the Study step, where outcomes are monitored to test the validity of the plan for signs of progress and success, or problems and areas for improvement. The Act step closes the cycle, integrating the learning generated by the entire process, which can be used to adjust the goal, change methods, reformulate a theory altogether, or broaden the learning – improvement cycle from a small-scale experiment to a larger implementation Plan. These four steps can be repeated over and over as part of a never-ending cycle of continual learning and improvement. | Multi-level | 20th century industry | Multiple applications | IOP |
| Quality Implementation Framework, Meyers, Am J Community Psychol, 2012 [28] | Four phases and fourteen steps.  Phase One: Initial considerations regarding the host setting  Assessment strategies  1. Conducting a needs and resources assessment  2. Conducting a fit assessment  3. Conducting a capacity/readiness assessment  Decisions about adaptation  4. Possibility for adaptation  Capacity-building strategies  5. Obtaining explicit buy-in from critical stakeholders and  fostering a supportive community/organizational climate  6. Building general/organizational capacity  7. Staff recruitment/maintenance  8. Effective pre-innovation staff training  Phase Two: Creating a structure for implementation  Structural features for implementation  9. Creating implementation teams  10. Developing an implementation plan  Phase Three: Ongoing structure once implementation begins  Ongoing implementation support strategies  11. Technical assistance/coaching/supervision  12. Process evaluation  13. Supportive feedback mechanism  Phase Four: Improving future applications  14. Learning from experience | Multi-level | Clinic, outpatient, multi-context not just healthcare, implementation research | Multiple applications | IO |
| The Translational Model of the Black Dog Institute, Werner-Seidler A, Frontiers in Psychology, 2016 [51] | Development of an Institute-specific translational model, the purpose of which is to guide staff in recognizing how their role contributes to the translational program, and to create a framework for translation. Pinwheel of six circles around edge connected to centre by spokes all bidirectionally connected. Knowledge creation information and resources radiate from centre to edges and outwards. Components include knowledge creation in the centre. Then a cycle of identify problem, select and adapt knowledge, implement programs, monitor knowledge use, evaluate outcomes, sustainable knowledge use. | multi-level (community-based and organizational) | Mental Health/Black Dog Institute Australia | Two examples provided where model is used Headstrong and a systems approach to suicide prevention. | IOP |
| Western Australia (WA) Health Network Policy Development and Implementation Cycle, Briggs AM, BMC Health Services Research, 2012 [30] | The aim of the WA Health Network is to involve all stakeholders with a shared interest in health to interact and exchange information with a view to collaboratively plan and facilitate implementation of consumer-centred (i) development of the Model of Care, (ii) policy uptake, and (iii) policy implementation. At each stage, stakeholder engagement, through engagement and consultation, is critical health services and policies (locally, coined “connect, share, improve”); therefore, this model may also be considered a community of practice approach. | Multi-level | Chronic disease, service delivery | Western Health Network model applied to musculoskeletal health. | IOP |

*I* individual, *O* organization, *P* policy
