# Supplementary material for: A scoping review of full-spectrum knowledge translation theories, models, and frameworks
Source: Implement Sci. 2020 Feb 14;15:11. doi: 10.1186/s13012-020-0964-5 (PMC7023795; doi:10.1186/s13012-020-0964-5)
Supplement: Supplementary file 3 — Additional file 3: Table S3. Full-spectrum KT theories, models, and frameworks that fit the Classic Theories Category (n = 8) [file 13012_2020_964_MOESM3_ESM.docx]

Additional file 3: Table S3 Full-spectrum KT theories, models, and frameworks that fit the Classic Theories Category (*n* = 8)

| Name of KT theory, model or framework, author, source, year | Description | Primary target audience or user | Context | How has it been used? | Level of use (I, O, P) |
| --- | --- | --- | --- | --- | --- |
| Diffusion of Innovations, Rogers, Diffusion of Innovations, 1983 [31] | Four elements in the diffusion of innovations: the innovation, communication channels, time and a social system. The innovation-decision process is the process through which an individual (or other decision-making unit) passes from first knowledge of an innovation to forming an attitude toward the innovation, to a decision to adopt or reject, to implementation of the new idea, and to confirmation of this decision. Five main steps in the innovation-decision process: (1) knowledge, (2) persuasion, (3) decision, (4) implementation, and (5) confirmation. | Multi-level | Diffusion research | Case illustrations presented; multiple examples in chapter and later articles. | IOP |
| Interorganizational Relations Theory, Steckler A book chapter, Mobilizing organizations for health enhancement: theories of organizational change. In: Glanz K, Rimer B, Lewis FM, eds. Health Behavior and Health Education: Theory, Research and Practice. 3rd ed. San Francisco, CA: Jossey-Bass; 2002:335–360  2002 and used website. <http://www.med.upenn.edu/hbhe4/part4-ch15-interorganizational-relations-theory.shtml> [33] | Interorganizational theory is a branch of organizational theory that focuses on how organizations work together. It is directed at the social ecology of multiple organizations that operate within communities. Collaboration among organizations often leads to more comprehensive and coordinated approach to public health than can be achieved by one organization. Components: Addresses change across organizations; Focuses on how organizations work together; Based on the premise that collaboration among community organizations leads to a more Comprehensive coordinated approach to a complex issue that can be achieved by one organization. | Organization/policy | Organizational research and industry | Grassroots coalitions of leaders from rural black churches, multiple hospital systems that reduce competition and provide flexibility in the face of accelerated changes in technology. | OP |
| Precaution Adoption Process Model (PAPM), The Precaution Adoption Process Model” by Neil D. Weinstein, Peter M. Sandman, and Susan J. Blalock, in Karen Glanz, Barbara K. Rimer, and K. Viswanath (eds.), Health Behavior and Health Education, 4th. ed. (San Francisco: Jossey-Bass, 2008), pp. 123–147 [35] | It is a stage theory. The PAPM attempts to explain how a person comes to decisions to take action and how he or she translates that decision into action. PAPM identifies seven stages along the path from lack of awareness to action. At some initial point in time, people are unaware of the health issue (Stage 1). When they first learn something about the issue, they are no longer unaware, but they are not yet engaged by it either (Stage 2). People who reach the decision-making stage (Stage 3) have become engaged by the issue and are considering tier response. This decision-making process can result in one of three outcomes: They may suspend judgment, remaining in Stage 3 for the moment. They may decide to take no action, moving to Stage 4 and halting the precaution adoption process, at least for the time being. Or, they may decide to adopt the precaution, moving to Stage 5. For those who decide to adopt the precaution, the next step is to initiate the behaviour (Stage 6). A seventh stage, if relevant, indicates that the behaviour has been maintained over time (Stage 7). | Clinic, community, patients, home, mass media, clinical, individual | Community-based, health behaviour research | Home radon testing and taking calcium for osteoporosis prevention but used theoretically not in practice. | IO |
| Self-Regulation Theory, Baumeister R, book chapter, Baumeister, Roy; Schmeichel, Brandon; Vohs, Kathleen. “Self-Regulation and the Executive Function: The Self as Controlling Agent”. Social psychology: Handbook of basic principles.  Self-regulation, ego depletion, and inhibition 2011 [34] | The activities and functions of the self, as well as the accumulated knowledge and understanding arising from research on the self, can be broadly grouped according to three main dimensions. These are presumably based on three basic phenomena that give rise to selfhood. The first is reflexive awareness: consciousness can be directed toward its source, so that just as people become aware of and learn about the world, they can also become aware of and learn about themselves. The eventual upshot is a body of knowledge and belief about the self, often called the self-concept. Without this, a self would be inconceivable. Second, the self is used to relate to others. People do not in fact develop elaborate self-concepts simply by contemplating themselves or reflecting on what they have done. Instead, they come to know themselves by interacting with others. The third is executive function or agent. The self exerts control over it environment by doing (making decisions and choices). | Individual | Philosophy of human nature | Unclear | I |
| Social Cognitive Theory (SCT), Bandura A, website: Bandura, Albert (1991). “Social Cognitive Theory of Self-Regulation” (PDF). Organizational Behavior and Human Decision Processes. <http://sphweb.bumc.bu.edu/otlt/MPH-Modules/SB/BehavioralChangeTheories/BehavioralChangeTheories5.html> [32] | It posits that learning occurs in a social context with a dynamic and reciprocal interaction of the person, environment, and behaviour. The unique feature of SCT is the emphasis on social influence and its emphasis on external and internal social reinforcement. SCT considers the unique way in which individuals acquire and maintain behaviour, while also considering the social environment in which individuals perform the behaviour. The theory takes into account a person’s past experiences, which factor into whether behavioural action will occur. These past experiences influences reinforcements, expectations, and expectancies, all of which shape whether a person will engage in a specific behaviour and the reasons why a person engages in that behaviour. The first five constructs were developed as part of the SLT; the construct of self-efficacy was added when the theory evolved into SCT.  1. Reciprocal Determinism - This is the central concept of SCT. This refers to the dynamic and reciprocal interaction of person (individual with a set of learned experiences), environment (external social context), and behavior (responses to stimuli to achieve goals).  2.Behavioral Capability - This refers to a person's actual ability to perform a behavior through essential knowledge and skills. In order to successfully perform a behavior, a person must know what to do and how to do it. People learn from the consequences of their behavior, which also affects the environment in which they live.  3.Observational Learning - This asserts that people can witness and observe a behavior conducted by others, and then reproduce those actions. This is often exhibited through "modeling" of behaviors. If individuals see successful demonstration of a behavior, they can also complete the behavior successfully.  4.Reinforcements - This refers to the internal or external responses to a person's behavior that affect the likelihood of continuing or discontinuing the behavior. Reinforcements can be self-initiated or in the environment, and reinforcements can be positive or negative. This is the construct of SCT that most closely ties to the reciprocal relationship between behavior and environment.  5.Expectations - This refers to the anticipated consequences of a person's behavior. Outcome expectations can be health-related or not health-related. People anticipate the consequences of their actions before engaging in the behavior, and these anticipated consequences can influence successful completion of the behavior. Expectations derive largely from previous experience. While expectancies also derive from previous experience, expectancies focus on the value that is placed on the outcome and are subjective to the individual.  6.Self-efficacy - This refers to the level of a person's confidence in his or her ability to successfully perform a behavior. Self-efficacy is unique to SCT although other theories have added this construct at later dates, such as the Theory of Planned Behavior. Self-efficacy is influenced by a person's specific capabilities and other individual factors, as well as by environmental factors (barriers and facilitators). | Individual | Philosophy of human nature | Unclear | I |
| Social Ecology Model for Health Promotion, Stokols D, American Psychologist, 1992 [37] | Dimensions and criteria for health-promotive environments: physical health, mental and emotional well-being, social cohesion at organizational and community levels. | Multi-level | Health promotion, social ecology in healthcare | Smoking and protect natural resources and quality of public environments. | IOP |
| Social Learning Theory (SLT), Bandura A, website, <https://www.betterhelp.com/advice/psychologists/albert-banduras-social-learning-theory> 1952 [36] | Development of a theory to explain learning behaviours/processes. All learning is the result of observing and modeling the behaviors of others. See Social Cognitive theory above.  Here are the stages of learning, according to Social Learning Theory.  1.Attention  2.Retention  3.Reproduction  4.Motivation | Individual | Psychology of learning | Unclear | I |
| Transtheoretical Model of Behaviour Change, Prochaska, JO, American Journal of Health Promotion, 1997 [39] | Uses a temporal dimension, the stages of change, to integrate processes and principles of change from different theories of intervention. The goal was a systematic integration of a field that had fragmented into more than 300 theories of psychotherapy. The transtheoretical model posits that health behavior change involves progress through six stages of change: precontemplation, contemplation, preparation, action, maintenance, and termination. Ten processes of change have been identified for producing progress along with decisional balance, self-efficacy, and temptations | Multi-level | Health promotion/smoking cessation intervention | Applied to smoking cessation interventions. | IO |

*I* individual, *O* organization, *P* policy
