# Supplementary material for: A scoping review of full-spectrum knowledge translation theories, models, and frameworks
Source: Implement Sci. 2020 Feb 14;15:11. doi: 10.1186/s13012-020-0964-5 (PMC7023795; doi:10.1186/s13012-020-0964-5)
Supplement: Supplementary file 4 — Additional file 4: Table S4. Full-spectrum KT theories, models, and frameworks that fit the Determinant or Evaluation Frameworks Category (n = 6) [file 13012_2020_964_MOESM4_ESM.docx]

Additional file 4: Table S4 Full-spectrum KT theories, models, and frameworks that fit the Determinant or Evaluation Frameworks Category (*n* = 6)

| Name of KT theory, model, or framework, author, source, year | Description | Primary target audience or user | Context | How has it been used? | Level of use (I, O, P) |
| --- | --- | --- | --- | --- | --- |
| Determinant frameworks (*n* = 3) | | | | | |
| Social Marketing Framework, Social Marketing National Excellence Collaborative, Turning Point National Program Office at the University of Washington, 2003Social Marketing and Public Health Lessons from the Field – SSWM <https://www.sswm.info/node/1984> [29] | There are three components that are essential to any definition. First is the role of marketing techniques—which necessitate putting the primary audience or target audience (aka “customer”) at the centre of every decision. Second is that the focus of the endeavour is on voluntary behavior change. Third, is that the behavior change is for the benefit of an individual, group, or population, not for profit or commercial gain. Social marketing integrates the marketing mix of the 5 Ps (Product, Price, Place, Promotion, and Policy factors) as well as the exchange and competition factors with the outcome of behavior change. | Multi-level | Public health | Twelve case studies described. | IOP |
| Consolidated Framework for Implementation Research (CFIR), Damschroder L, Implementation Science, 2009 [40] | The CFIR is “meta-theoretical”. It includes constructs from a synthesis of existing theories, without depicting interrelationships, specific ecological levels, or specific hypotheses. The CFIR is composed of five major domains: intervention characteristics, outer setting, inner setting, characteristics of the individuals involved, and the process of implementation. Eight constructs were identified related to the intervention (e.g. evidence strength and quality), four constructs were identified related to outer setting (e.g. patient needs and resources), 12 constructs were identified related to inner setting (e.g. culture, leadership engagement), five constructs were identified related to individual characteristics, and eight constructs were identified related to process (e.g. plan, evaluate, and reflect). | Multi-level | No specific context, consolidating multiple framework constructs | Multiple application | IOP |
| Knowledge Integration Process, Glasgow RE, Am J of Public Health, 2012 [54] | There are 5 overlapping, interrelated phases of research in advancing from scientific discoveries to population health. Whatever terms are used for these phases and whether public health or medical issues are under consideration, the diagram illustrates the highly iterative nature of the cycle from discovery to translation. The process starts with the identification of a problem and the “discovery” of an opportunity or approach to tackle a health issue (T0). These discoveries can result from multiple sources and disciplines such as molecular or biological insights, behavioral research, or epidemiological research. The first translational research phase (T1) involves research allowing for the development of tests or other clinical interventions, but it can also lead to nonmedical interventions such as policy, behavioral, social, or other public health interventions. The second research phase (T2) involves a rigorous analysis and investigation of whether the new interventions improve health outcomes (in randomized trials or other study designs). The end result of T2 is evidence-based guidelines and recommendations by professional organizations and independent panels. (National Institutes of Health often focuses primarily on T1 and T2 research, usually in the context of drug and other clinical interventions and incorporates all T2–T4 activities under T2). | Multi-level | Dissemination research, NIH, Public health | Lorig et al. program implementation and other examples in paper. | IOP |
| Evaluation frameworks (*n* = 3) | | | | | |
| A Conceptual Framework for Planning and Improving Evidence-Based Practices, Spencer, Prev Chron Disease, 2013 [53] | The workgroup produced a conceptual framework consisting of two interrelated components: public health impact and quality of evidence. The public health impact component of the framework consists of the following 5 elements: effectiveness, reach, feasibility, sustainability, and transferability. These elements are derived in part from the reach, effectiveness, adoption, implementation, maintenance (RE-AIM) model for translational research [41]; the integrative validity model; and the systematic screening and assessment method. The quality-of-evidence component refers to where a practice lies on an evidence-based practice continuum. These elements represent four levels of evidentiary quality—weak, moderate, strong, and rigorous. At the intersection of impact and quality of evidence, a continuum of evidence-based practice emerges. This continuum represents the ongoing application of knowledge about what is working to strengthen impact in a given context. A continuum was created consisting of 4 stages—emerging, promising, leading, and best. | Multi-level | Public Health Translational Research | Center for Disease Control programs | IOP |
| PRECEDE-PROCEED, Green, book chapter, Green L, Kreuter M. Health Program Planning: An Educational and Ecological Approach. 4th edition ed. McGraw-Hill Higher Education; New York, NY: 2005 [42] | The framework has two components: the first set of phases consists of a series of planned assessments that generate information that will be used to guide subsequent decisions. This series of phases involves considerable sifting and sorting and is referred to as PRECEDE for predisposing, reinforcing, and enabling constructs in educational/ecological diagnosis and evaluation. The second component is marked by the strategic implementation of multiple actions based on what was learned from the assessments in the initial phase. The second component is named PROCEED for policy, regulatory, and organizational constructs in educational and environmental development. Evaluation is an integral part of both phases and serves as the primary vehicle to ensure the quality of the planning process. | Multi-level (practitioner, epidemiologists, social, behavioural, economic, and political scientists) | Health research | Used to guide planning efforts addressing health issues in communities, schools, health care settings and workplaces. | IOP |
| RE-AIM, Glasgow, America Journal of Public Health,1999 [41] | It is an evaluation framework for public health. The public health impact of an intervention as a function of 5 factors: reach, efficacy, adoption, implementation, and maintenance. Each of the 5 RE-AIM dimensions is represented on a 0 to 1 (or 0 to 100%) scale. | Individual or organizational | Healthcare  Public health | Multiple applications | IO |

*I* individual, *O* organization, *P* policy
