# Supplementary material for: A scoping review of full-spectrum knowledge translation theories, models, and frameworks
Source: Implement Sci. 2020 Feb 14;15:11. doi: 10.1186/s13012-020-0964-5 (PMC7023795; doi:10.1186/s13012-020-0964-5)
Supplement: Supplementary file 5 — Additional file 5: Table S5. Full-spectrum KT theories, models, and frameworks that fit More than one Theoretical Approach Category (n = 4) [file 13012_2020_964_MOESM5_ESM.docx]

Additional file 5: Table S5 Full-spectrum KT theories, models, and frameworks that fit More than one Theoretical Approach Category (*n* = 4)

| Name of KT theory, model, or framework, author, source, year | Description | Primary target audience or user | Context | How has it been used? | Level of use (I, O, P) |  |
| --- | --- | --- | --- | --- | --- | --- |
| Process Model and Classic Theory (*n* = 1) | | | | | |  |
| Stage Theory of Organizational Change, Butterfoss FD, book chapter, Karen Glanz, Barbara K. Rimer, and K. Viswanath, 2008 Health Behavior and Health Education: Theory, Research, and Practice, 4^th^ Edition [38] | According to stage theory of organizational change, adoption of an innovation by an organization typically follows several stages. Each stage requires a specific set of strategies that are contingent on the organization’s stage of adoption, implementing, and sustaining new approaches, as well as on the socioenvironmental factors that may be outside the organization’s control. To use stage theory effectively, the social environment and the innovation’s stage of development must be carefully assessed before appropriate strategies are selected for each stage. 1. Define problem (awareness stage); 2. Initiate action (adoption stage); 3. Implement change; 4. Institutionalize change. | Organization/policy | Organization change research/industry | Unclear | OP |  |
| Classic Theory and Determinant Framework (*n* = 2) | | | | | | |
| Community Connection Model, Liddy C, Public Health, 2013 [43] | This model of program implementation and sustainability consists of four components policy, partnership, planning and program that interact at all stages of program development and implementation. The Community Connection Model demonstrates how sustainable programs can “be grown” by establishing and maintaining interaction between the four components of policy, partnership, planning and program. | Multi-level | Chronic disease | Tested the Stanford Chronic Disease Self-Management Program; model developed. living health Champlain workshop. | IOP |  |
| Community to Community Mentoring (CCM) model, Delafield R, Progress in Community Health Partnerships: Research, Education and Action, 2016 [44] | The CCM model uses Community-based participatory research (CBPR) principles to orient an approach to dissemination with two objectives: (1) effectively disseminating Evidence-based interventions (EBI)s while (2) building organizational and community capacity to create sustainable changes required to improve the health and well-being of participating communities. Specifically, the CCM model combines elements of the Diffusion of Innovations Theory, the social cognitive theory, and key concepts that are common to community organizing and community building processes and practices. The CCM model uses a CBPR approach applied to EBI dissemination research. It does this through nesting the dissemination of innovations (in this case the EBI) within a CBPR orientation. There are four major components of the CCM model: (1) the context, (2) the CBPR principles, (3) the partnerships, and (4) the innovation elements. | Multi-level | Community | It was used for the pilot study of five new community partners. | IOP |  |
| Process Model and Evaluation Framework (*n* = 1) | | | | | |  |
| Evidence-Driven Community Health Improvement Process (EDCHIP), Layde, American Journal of Public Health 2012 [52] | This model started with the framework of the Community Health Improvement Process (CHIP). To incorporate current thinking on evidence-based public health interventions, this model modifies CHIP’s planning and evaluation phases. Elements of evidence-based public health framework are incorporated into planning phase to make explicit the need to search the scientific literature and organize information. In addition, communicating to key community stakeholders’ critical information on community patterns of disease and risk factors and on efficacious interventions identified from the literature. For evaluation, the reach, effectiveness, adoption, implementation, and maintenance (RE-AIM) model of Glasgow et al. is followed which has been used in translation research [41]. | Community | Public health, community-based research | Community-based applications | IOP |  |

*I* individual, *O* organization, *P* policy
